# A Software Framework for Intelligent Security Measures Regarding Sensor Data in the Context of Ambient Assisted Technology

**DOI:** 10.3390/s23146564

**Published:** 2023-07-20

**Authors:** Shakeel Ahmed, Parvathaneni Naga Srinivasu, Abdulaziz Alhumam

**Affiliations:** 1 Department of Computer Science, College of Computer Sciences and Information Technology, King Faisal University, Al-Ahsa 31982, Saudi Arabia; aahumam@kfu.edu.sa; 2 Department of Computer Science and Engineering, Prasad V Potluri Siddhartha Institute of Technology, Vijayawada 520007, India; parvathanenins@gmail.com

**Keywords:** ambient assistive technology, encryption, Internet of Medical Things, security framework, residual energy, energy consumption, network lifetime

## Abstract

Ambient assisted technology (AAT), which has the potential to enhance patient care and productivity and save costs, has emerged as a strategic goal for developing e-healthcare in the future. However, since the healthcare sensor must be interconnected with other systems at different network tiers, distant enemies have additional options to attack. Data and resources integrated into the AAT are vulnerable to security risks that might compromise privacy, integrity, and availability. The gadgets and network sensor devices are layered with clinical data since they save personal information such as patients’ names, addresses, and medical histories. Considering the volume of data, it is difficult to ensure its confidentiality and security. As sensing devices are deployed over a wider region, protecting the privacy of the collected data becomes more difficult. The current study proposes a lightweight security mechanism to ensure the data’s confidentiality and integrity of the data in ambient-assisted technology. In the current study, the data are encrypted by the master node with adequate residual energy, and the master node is responsible for encrypting the data using the data aggregation model using a node’s key generated using an exclusive basis system and a Chinese remainder theorem. The integrity of the data is evaluated using the hash function at each intermediate node. The current study defines the design model’s layered architecture and layer-wise services. The model is further analyzed using various evaluation metrics, such as energy consumption, network delay, network overhead, time in generating hash, tradeoff between encryption and decryption, and entropy metrics. The model is shown to adequately perform on all measures considered in the analysis.

## 1. Introduction

The purpose of ambient assisted living (AAL) technology [1] is to make people safer in their homes by alerting them to potential risks before they occur. Indeed, the goal of AAL is to foster innovation to keep individuals connected, healthy, active, and satisfied as they age. AAL covers deploying and applying intelligent technology to help the elderly deal with challenges and live in their chosen setting for longer. AAL employs information technology to monitor the health and wellbeing of the elderly, reducing the human resources necessary to care for them. Elderly and frail people, in particular, may considerably benefit from the possibilities afforded by assistive technology in their privacy settings, boosting their health-related protection and preserving overall physiological wellbeing. Ambient assisted living technology reduces human resources concerning caretakers, making real-time monitoring economically feasible, effective disease handling, deep surveillance, and activity monitoring technology. 

AAT technology comprises divergent devices ranging from real-time activity sensors to warehouse management. The security of sensitive data over divergent devices is exceedingly challenging to monitor and manage. A lightweight data encryption algorithm that would be deployed in the energy-centric environment is desired to ensure the confidentiality of the data. Although AAT makes our lives safer and health more effective, the existing issues of expanding data exchange, bandwidth deficiency, high latency, and limited mobility support of the sensors in technology exacerbate these limitations that impede the pervasive adoption and implementation of novel technologies [2]. Furthermore, other difficulties remain unresolved, such as privacy and security concerns, location awareness, centralized data storage and processing, high data processing delays, network congestion, and communication costs [3]. The current study focuses on addressing a few of the issues mentioned above in handling and processing the real-time sensor data of the users. According to a report, the healthcare security industry alone will be worth $8.7 billion by 2023 [4].

**Figure 1:**
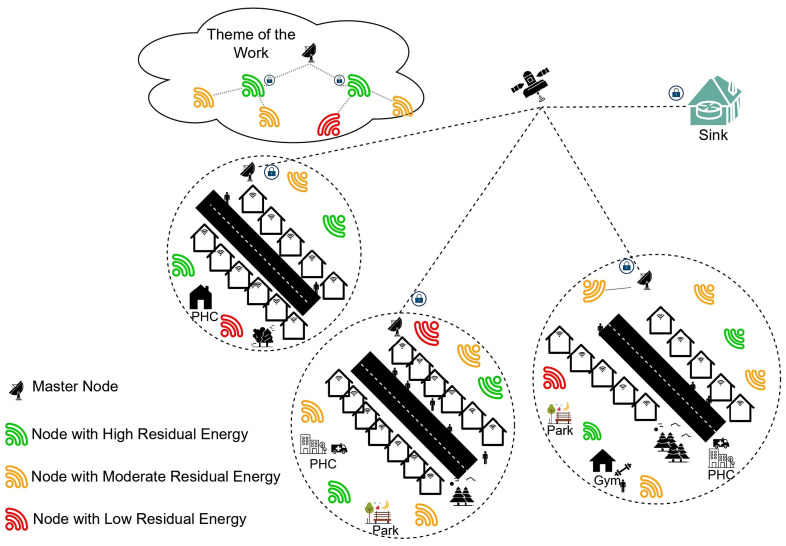
Ambient assisted environment with interconnected sensor nodes for data exchange.

Many approaches are utilized to ensure the privacy of data transmitted through sensor devices within the Internet of Medical Things (IoMT) [5]. Some of the approaches include public key-based data encryption [6], blockchain technology [7,8], Deep Learning for encryption [9], and the data aggregation mechanism [10]. As a crucial data processing method, data aggregation may minimize energy and bandwidth usage while gaining correct information by integrating message authentication codes with each data packet rather than sending an auxiliary packet with checksum and error detection codes [11]. Although data aggregation security mechanisms enhance the confidentiality of the data exchanged across the edge devices in IoT, attackers can eavesdrop on conversations between organizations, tamper with the original messages, and forge digital signatures. As a result, the legitimacy of aggregated data is uncertain, and the cloud center’s decision may be jeopardized. As a result, privacy-preserving aggregated data have arisen as an essential research topic. Additionally, healthcare services for customers are at risk of disruption and data loss due to ransomware attacks on private data operations hosted on public servers [12]. The sensors in the ambient environment settings are shown in Figure 1. 

The motivation of the current study is the security concern associated with connections among AAT devices and sensors that must be safe and constantly accessible because of the sensitive information being handled in ambient assisted living. Furthermore, data integrity, confidentiality, and availability are critical for medical data exchanged through the ambient sensors, as well as base stations in the hospital’s network. Despite the new technologies providing opportunities for AAT’s advancement and growth, they pose various security and privacy concerns that may impair the deployment and use of the ambient assisted living ecosystem [13]. These underlying challenges have largely motivated the current study’s design of the lightweight security model. The contributions of the current study are listed as follows.

- Reviewing various existing models ensures the confidentiality of the data in the AAT and IoMT environments.
- An energy-centric sensor device selection mechanism to identify the local and global host device that performs data encryption.
- Ensuring the confidentiality of the data being exchanged over the sensor devices in AAT technology.
- Evaluating the security model concerning various aspects of the performances, such as the throughput and latency, and confidentiality metrics, such as correlation and entropy of the encrypted data.
- Finally, presenting the open challenges in this field and future motivational trends.

The rest of the sections in the manuscript are organized on the following grounds. Section 2 presents various contemporary encryption techniques used in the IoT environment to maintain the privacy of the data being exchanged. Section 3 presents the background of the study, where the layered architecture and the services of each layer in the proposed software-driven security model are discussed, and also the metrics used in evaluating the performance. Section 4 presents the proposed encryption, key generation, and management methodology. Section 5 presents the experimental results and the statistical analysis with other contemporary models. Finally, Section 6 presents the study’s conclusion and future research directions. 

## 2. Literature Review

Although there are various interpretations of the ambient assisted living environment, a fundamental technological idea is to link sensors, smart wearable devices, and healthcare management applications over the internet to provide remote monitoring, access, and control of the environment. As a result, ambient environments seek to identify and offer personalized services to citizens who interact with and collaborate with the environment. An ambient assisted environment is also automated, using the Internet of Things (IoT) [14] framework, capable of responding to the needs of its occupants, offering comfort and wellbeing.

Several designs have been developed to bring security and privacy characteristics to IoT network operating situations. Hamed et al. [15] suggested a safe artificial intelligence (AI)-based framework for protecting an IoT edge layer. The Cyber Kill Chain model was used in this research to identify and categorize the life cycle of any threat. The many types of threats and how AI engines deal with them at the edge level were also assessed. A new safe IoT paradigm for managing supply chain risk was introduced [16]. Machine learning algorithms, cryptographic hardware monitoring, and distributed system coordination all assist in the architecture’s security. Alam et al. [17] proposed a layered architecture for the IoT to promote secure accessibility to IoT-enabled services and ensure compatibility of security characteristics across various administrative domains. Using a semantically enhanced overlay, they connected the various layers and ensured the platform’s security by employing ontology-based reasoning and semantic rules. Semantic criteria were designed to ensure access authorization, as this study is solely concerned with the security requirements of access control problems. Wang et al. [18] proposed a technique for anonymous aggregation in public cloud computing utilizing fog. Bilinear pairings and the Castagnos-Laguillaumie cryptosystem can be used to execute the calculation over ciphertexts. Data privacy between the information producer and the aggregator and data compression between the aggregator and the receiver, or base station, were two key motivations for these information-based methods [19]. However, extensive computing efforts are required for the suggested aggregation model using the Castagnos-Laguillaumie cryptosystem. Zhao et al. [20] have suggested an effective WBAN authentication scheme without bilinear pairing operations. Elliptic-Curve Cryptography (ECC) was used for identity-based authentication for wireless body area networks (WBAN). ID-based communication does not need a certificate. Zhao’s approach is unsafe because attackers may track the user using the pseudo identity’s constant value.

The recent study by Loretta and Kavitha [21] integrated the best clustering technique into the model to protect data privacy. This protocol improved energy-efficient and data-privacy routing across the heterogeneous network by using multi-hop transmission and clustering to reduce sensor node energy consumption and increase network lifespan. The simulations showed that the suggested technique improved data security while considering the network lifespan and computing time. Blockchain technology is the other most widely used technology to ensure the confidentiality of the data in the IoT. The blockchain-based privacy-preserving approach employs the blockchain technology concept. The basic concept is a decentralized, distributed ledger containing encrypted data chunks. The data block in the blockchain is made up of a sequence of transactions that most network members have approved. For unique identification, the blocks are chronologically connected using the prior hash value for the next consecutive block. The associated hash code of the block may be used to locate the block in the blockchain. These connected blocks must be distributed and copied in the peer-to-peer network. The distributed consensus technique inserts transactions into new blocks and spreads consensus (evidence) throughout the network. Creating blocks in the network is carried out by distinct nodes, notably miners.

The current study primarily focuses on medical device data and healthcare application computations over the edge devices. High-risk patients with post-diagnosis side effects need prompt decisions from healthcare systems. Due to the considerable computing and storage resources, numerous researchers use the cloud layer for computations. Latency in sending Cloud findings to devices hinders diagnosis for people who need immediate decisions. Medical information processing and information interchange in healthcare services increase Cloud-IoT channel communication overhead. Data analysis at the edge layer, including the sink, reduces latency, bandwidth, and network delay to allow real-time applications. Multi-Access Edge Computing (MAEC) [22] also prevents network bottlenecks and congestion on the lengthy transmission channel between the interface layer and the data processing layer.

## 3. Background

**Figure 2:**
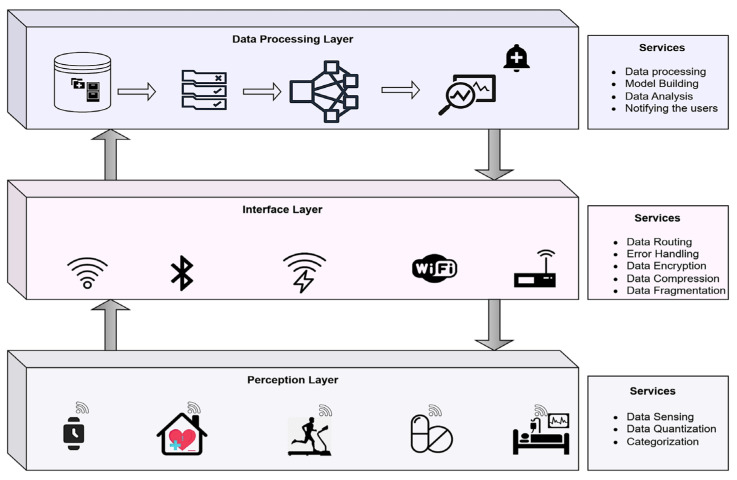
The layered framework of the proposed network model.

The AAT network’s installed sensors would use the IoMT framework to collect and share data. In a real-time setting, people and patients are constantly watched, with information flowing back and forth between sensor nodes and a central hub. The network model distributes roles and services across its tiers to make processing data more manageable. In Figure 2, we see the three levels of the network and their respective roles: the perception layer, the interface layer, and the data processing layer. 

*Perception Layer:* The perception layer is the bottommost layer of the proposed model, which generally comprises the sensor devices deployed in the AAL environment that range from wearable sensors to sensors deployed at smart homes for real-time monitoring of the patients and the citizens. This layer offers services such as data acquisition from sensor devices, quantization, and categorical data handling. The raw data is acquired from the sensors and processed as perception data. 

*Interface Layer:* The interface layer is the intermediate layer in the proposed framework, where the interface layer deals with multiple network technologies such as Bluetooth, Wi-fi, ZigBee, and Z-wave technologies used in exchanging data. This layer offers services such as data routing between the base station and the sensor devices and error handling, and other crucial services, such as data compression and encryption, are carried out in this layer. 

*Data Processing Layer:* The data processing layer is the topmost layer that generally focuses on building a model that can analyze the data and notify the user during abnormal situations. The layer comprises the database to maintain the sensor data, and the database would be the source of input for the model to analyze the data. Furthermore, the abnormal medical parameters would trigger the notification to the patients about their health condition. 

All the encryption operations will occur in the interface layer of the network architecture discussed in the current study. The proposed software-defined network would be part of the interface layer of the proposed layered framework. The current study is limited to security-related services of the interface layer. 

### 3.1. Metrics for Evaluation

Various parameters are considered in evaluating the privacy and confidentiality-related aspects of the data exchanges over the sensor nodes. Some metrics include network delay, computational time, energy consumption, and system reliability. The mathematical equations used in evaluating these metrics are discussed as follows. 

*Network Delay:* The network delay for Internet of Things applications consists of round-trip delays, which include the delay between the slave node $S_{n}$ and the corresponding sink $S_{k}$ and the corresponding master node $M_{n}$. The notation $S_{n}↔M_{n}$ denotes the round-trip time between the slave node and the master node, and similarly, the notation $M_{n}↔Sk$ designates the round-trip time between the sink and the master. Many factors, including noise, inference, and interruptions in the wireless network, can disrupt links to communication throughout task offloading and downloading. As a result, the offloading judgment approach considers dynamic changes in network circumstances during offloading [23,24]. The formula for the network delay is calculated based on the delays incurred by all the other factors, as discussed above. The network delay at master nodes is shown in Equation (1), and the delay at the sink is measured as shown in Equation (2) [25].

(1) $$N_{d}\left(M_{n}\right)=\left\{\alpha \times \left(M_{n}\right)+\beta \times \left(M_{n}\right)\right\}\times C+\langle S_{n}↔M_{n}\rangle log_{2}\left(1+\frac{S_{p}}{C_{n}}\right)$$

(2) $$N_{d}\left(S_{k}\right)=\left\{\beta \times \left(S_{k}\right)\right\}\times C+\langle M_{n}↔S_{k}\rangle log_{2}\left(1+\frac{S_{p}}{C_{n}}\right)$$

From the above equations, the variables α and β represent the download and upload tasks, respectively. The notation C designates the total channel capacity, including the delays of all upload and download tasks. The notation $S_{p}$ designates the network’s signal power measured in watts and the notation $C_{n}$ designates the noise power in the corresponding channel, which is considered to be 15 dB across the experimentation. 

*Computational time:* The computational time is crucial in assessing the time consumed at the master nodes for aggregating the data they have received from the slave nodes. It is approximated as the sum of the queuing delay $\left(q_{d}\right)$, processing delay $\left(p_{d}\right),$ and the delay associated with download and upload designated by $n_{dl}$ and $n_{ul}$, respectively. This is generally desired to be low for a network with better performance. The computation delay and the network delay are hard to normalize. Hence, the delay is calculated as a combination of the computational and network delays. The formula for an overall computational time at the master node and sink is shown in Equations (3) and (4), respectively.

(3) $$C_{t}\left(M_{n}\right)=\{n_{dl}+q_{d}+p_{d}+n_{ul}\}+N_{d}\left(M_{n}\right)$$

(4) $$C_{t}\left(M_{n}\right)=\{n_{dl}+q_{d}+p_{d}\}+N_{d}\left(S_{k}\right)$$

*Energy consumption:* The other most significant parameter considered in evaluating the network model is the computational delay, which is generally desired at a minimum [26]. The formulas for the computational delay at the master node and sink are presented in Equations (5) and (6), respectively [27]. The overall computational delay at the network is shown in Equation (7).

(5) $$C_{t}\left(M_{n}\right)=t_{sn}\times \left\{B_{l}\times D_{af}\right\}\times amp\times d^{2}$$

(6) $$C_{t}\left(S_{k}\right)=t_{mn}\times \left\{B_{l}\times D_{af}\right\}\times amp\times d^{4}$$

(7) $$C_{t}\left(Overall\right)=C_{t}\left(M_{n}\right)+C_{t}\left(S_{k}\right)$$

From the above Equations, the notation $C_{t}$ designates the computational time for the exchange of $B_{l}$ length of bits in the network. The notation $D_{af}$ designates the data aggregation factor, and the notation amp designates the amplifier to retain the signal-to-noise ratio in the communication. The notation $t_{sn}$ designates the sum of all active slave nodes in the network and $t_{mn}$ designates the sum of all master nodes in the network that are responsible for the exchange of the data in the associated cohort. 

### 3.2. Implementation Environment

The proposed model was simulated in a Network Simulator 2 platform. A 100×100-m elevation grid was the platform for analyzing the proposed scenario. In this situation, transmissions might be detected up to a distance of 30 m. The model was built using a single repository that was run 2000 times. The security model was implemented through the cryptography library using the Fernet package. The network simulation and the encryption of the sensor data were performed as two independent processes, and the cumulative performance was evaluated. Table 1 presents the specifics of the simulation environment. 

### 3.3. Network Model 

**Figure 3:**
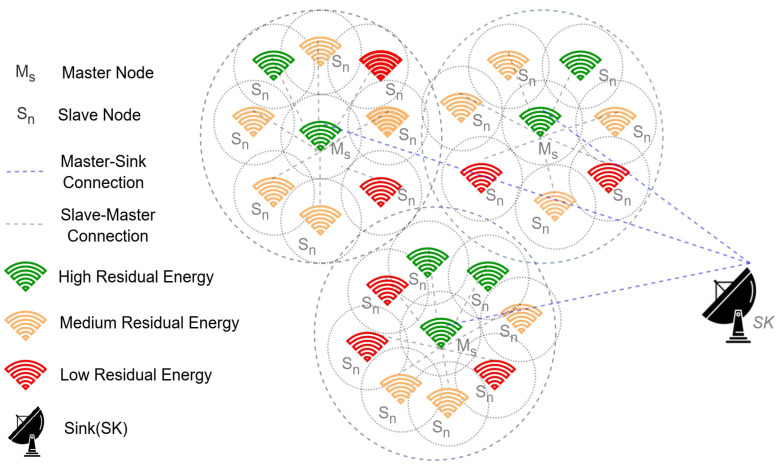
Network model of the proposed framework.

The proposed network consists of 100 nodes with divergent residual energy at initial deployment, and the nodes are grouped as a cohort that relies on the distance and likelihood of the nodes. All of the nodes in the cohort would rely on the node with the highest residual energy at the current round, named the master node, and the rest of the cohort nodes are considered slaves. The slaves send the sensor data to the corresponding master, and the master aggregates the data to ensure the data’s confidentiality. Every cohort in the network will have a master node, and all the master nodes are directly connected to the sink. The sink has a business model that performs data analytics and notifies users of any abnormality. The master node in the cohort is updated in successive rounds based on the available resources and other underlying factors. The grouping of network nodes and identification of the master nodes are discussed in the current sub-section. The proposed software-driven network model is shown in Figure 3.

#### 3.3.1. Grouping the Network Nodes

The node grouping is carried out to form a cohort of nodes in the network, where each cohort does have a master node that is responsible for exchanging the data. The process of forming the cohort of nodes is carried out based on the distance and the delay timer [28]. The cluster formation’s objective function is based on Equations (8) and (9).

(8) $$O_{f}=td_{min}+\left(td_{max}-td_{min}\right)\times \gamma \times \left(\frac{e_{d}}{r_{e}}\right)+td_{rand}$$

(9) $$e_{d}=\sqrt{{\left(y_{2}-y_{1}\right)}^{2}+{\left(x_{2}-x_{1}\right)}^{2}}$$

From the above equation, the notations $td_{max}$ and $td_{min}$ designate the maximum and minimum time delay associated with the nodes. The notation γ designates the non-dimensional energy and distance metrics. The notation $e_{d}$ designates the Euclidean distance approach and the notation $r_{e}\;$ designates the value of the residual energy. The $\;td_{rand}\;$ is a random delay timer value. The value of the random delay duration is selected to avoid several sensors simultaneously becoming the master. 

#### 3.3.2. Assessing the Residual Energy

The residual energy at the node is one of the crucial deciding factors for grouping the nodes, and the Master is partly determined by the amount of residual energy. The nodes are initialized with divergent residual energy, and the available residual energy is updated in each round to the node with the highest residual energy and the node at a feasible distance to the rest of the nodes in the cohort as the master. The residual energy is updated based on the formula shown in Equation (10).

(10) $$r_{e}=r_{e_{int}}-\left\{\left(\chi \times d\right)+\left(E_{n}\times d\times e_{d}\right)\right\}$$

From the above equation, the notation $r_{e_{int}}$ designates the initial residual energy, and the notation d designates the number of data packets. The notation χ designates the implied energy and $E_{n}$ designates the energy needed to push the data packets to the next node; i.e., when the node is master, the energy needed to push from the master to the sink, and when the node is slave, the energy needed to push the data from the slave to the master. 

#### 3.3.3. Assessing the Cohort Fitness

The group’s fitness is assessed to retain it in the next consecutive rounds. In case of fitness values less than 0.5, the nodes in the cohort are merged into the neighboring cohort based on the distance measured. The cohort’s fitness $c_{f}$ is assessed in the following Equation (11).

(11) $$c_{f}=\frac{1}{\sum_{i}^{t_{c}}\sum_{j}^{t_{n}}\sqrt{\left|n_{r_{e}}-m_{r_{e}}\right|}}$$

From the above equation, the notations $t_{c}$ and $t_{n}$ designate the count of cohorts and the count of nodes in the cohort, respectively. The notation $n_{r_{e}}$ denotes the residual energy at the corresponding node, and the $m_{r_{e}}$ is the mean residual energy at the corresponding cohort. 

## 4. Proposed Methodology

The data obtained by the cluster head is accumulatively encrypted through a lightweight encryption process, guaranteeing the confidentiality of the data exchanged between the master and the sink. Although the majority of data utilized in data communication applications are massive, only a tiny fraction of it is valuable in WSNs. WSNs commonly employ symmetric-key encryption techniques, whereby a large key size encrypts a substantial data block. The efficacy and durability of the encryption protocol are contingent upon the larger pairwise keys mutually shared by the entities involved in data transmission. The data accumulation procedure encrypts the data acquired at the master using the path-oriented encryption approach. The path-oriented encryption approach necessitates lower keys for encryption and resource efficiency, requiring less computing time and effort than other contemporary encryption methods. The suggested data accumulative model encrypts data using a series of arithmetic operations and numerous keys, ensuring the model’s confidentiality. The keys used for data encryption are randomly created, and the size of a key is proportionate to the data size. The proposed model is a lightweight encryption with fewer computations and rounds than conventional encryption models.

**Figure 4:**
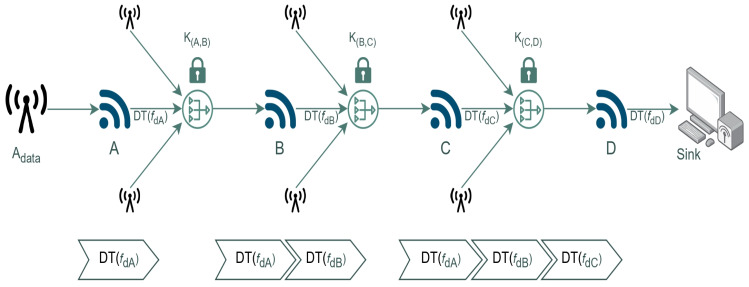
Accumulative data encryption model for the sensor data.

On the other hand, the key generation process is computationally feasible using the Chinese reminder theorem with simple modulus operation. The notations used in the current section are enclosed in Table 2. The smart security framework for the sensor data is shown in Figure 4. The notations used in the current section are shown in Table 2. 

### 4.1. Encryption and Decryption Process

The security model’s operation may be better understood by utilizing an example with four nodes, notably A,B,C, and D. In this scenario, it is presumed that node A is the origin of the data that transmits the data to node D. The nodes B and C are considered intermediate between the nodes A and D. The acquisition of sender data is facilitated by linking nodes, whereby each node shares a confidential key employed in the encryption process. The mathematical modeling for secured data communication can be understood through Equations (12) to (16).

(12) $$DT\left(f_{dA}\right)=A_{data}\times HV_{data}+\left\{k_{\left(A,B\right)}+k_{\left(A,C\right)}+k_{\left(A,D\right)}\right\}$$

(13) $$DT\left(f_{dB}\right)=DT\left(f_{dA}\right)-\left\{k_{A,B}\right\}+\left\{k_{B,C}+k_{C,D}\right\}$$

(14) $$DT\left(f_{dC}\right)=DT\left(f_{dB}\right)-\left\{k_{A,C}+k_{B,C}\right\}+\left\{k_{C,D}\right\}$$

(15) $$DT\left(f_{dD}\right)=DT\left(f_{dC}\right)-\left\{k_{A,D}+k_{B,D}+k_{C,D}\right\}$$

(16) $$A_{data}=\frac{DT\left(f_{dD}\right)}{HV_{data}}$$

As can be observed from the above equations, a basic arithmetic operation sequence is used for the full encryption and decryption. Whenever the data reaches the node downstream, the upstream key list is updated with the corresponding key. The sink would use the key at the final intermediate node to decrypt the cipher text. The keys for data encryption are generated using a key generator. The data hash is examined to confirm the data’s integrity. The predicted hash value is multiplied before transmitting the data. The same hash value is utilized at the receiving end to divide the generated data so that the data’s integrity may be correctly assessed. Assume the data have been altered since they cannot be decrypted due to the same hash value being multiplied by the original data.

### 4.2. Data Accumulation Process

**Figure 5:**
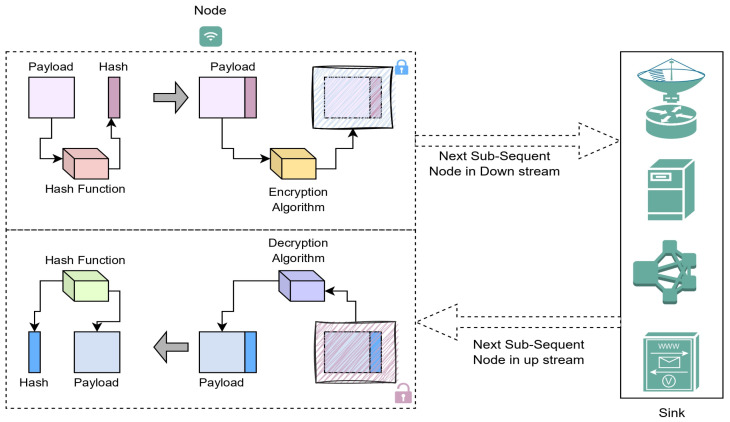
Data encryption and decryption process in the proposed network design.

The data accumulation is carried out at each node, the data of the current node are appended to the data from the upstream, and then, on appending the data to the received data, the data are transmitted to the node downstream. The recent data at the current sensor node are fed as the data packet and the input for the has function, and then, the assessed has value is appended to the data packet. Then, the data packet is encrypted using the key at the sensor device. The calculated hash value is used to assess the integrity of the data being exchanged. The data encryption process is shown in Figure 5 for a better understanding of the process. Accumulative data encryption is applied to data retrieved from numerous sources linked to the node. Equations (17) to (20) show the corresponding formulas for the data accumulation process.

(17) $$Acc\left(A\right)=A_{data}\times HV_{data}+\left\{A\}+\{k_{\left(A,B\right)}+k_{\left(A,C\right)}+k_{\left(A,D\right)}\right\}$$

(18) $$Acc\left(B\right)=\left\{Acc\left(A\right)+B\right\}-\left\{k_{A,B}\right\}+\left\{k_{B,C}+k_{C,D}\right\}$$

(19) $$Acc\left(C\right)=\left\{Acc\left(A\right)+Acc\left(B\right)+C\right\}-\left\{k_{A,C}+k_{B,C}\right\}+\left\{k_{C,D}\right\}$$

(20) $$A_{data}=\frac{Acc\left(A\right)+Acc\left(B\right)+Acc\left(C\right)}{HV_{data}}$$

The lightweight encryption mechanism is complex enough to assure data confidentiality. The activities are conducted at the cluster head to guarantee that the encryption process does not overburden the other linked sensor nodes, which would result in a more sustainable and robust network. 

### 4.3. Key Generation and Management

The key generation is one of the significant phases of the proposed security model, where the key’s strength decides the framework’s robustness in ensuring the confidentiality of the information being exchanged. The proposed key model relies on the Exclusive Basis System (EBS) and Chinese remainder theorem (CRT) [29]. The CRT is extensively used in cryptosystems as it deals with larger integers to address the modular congruence relations with different moduli. A 1024-bit key is generated using the proposed model used in the encryption process. The nodes in the proposed model will have k keys out of the total $\;t_{k}$ number of keys, where $\left\{k<t_{k}\right\}$ at all the instances. In all the instances, the cohort’s key is determined as shown in Equation (21).

(21) $$c_{key}=\left(E\_Acc_{data}\;mod\;\;I_{m}\right)+p_{key}$$

The accumulated data are encrypted using the CRT across multiple keys, as shown in Equation (22).

(22) $$\begin{array}{c} E\_Acc_{data}=key_{1}\;mod\;n_{key\_1}\\ E\_Acc_{data}=key_{2}\;mod\;n_{key\_2}\\ E\_Acc_{data}=key_{3}\;mod\;n_{key\_3}\\ .\\ .\\ E\_Acc_{data}=key_{k}\;mod\;n_{key\_k}\; \end{array}\}$$

The corresponding keys at each node are recognized as $\left\{n_{key\_1},n_{key\_2},n_{key\_3}\ldots n_{key\_k}\right\}$ used in encrypting the accumulated data at the node. CRT only holds when the $\;n_{key\_i}$, where $\left\{i=1,2\ldots ,k\right\},\;$are mutually co-prime. The node key is generated, as shown in Equation (23).

(23) $$n_{key\_i}=c_{key}\;m_{key}-p_{key}$$

**Figure 6:**
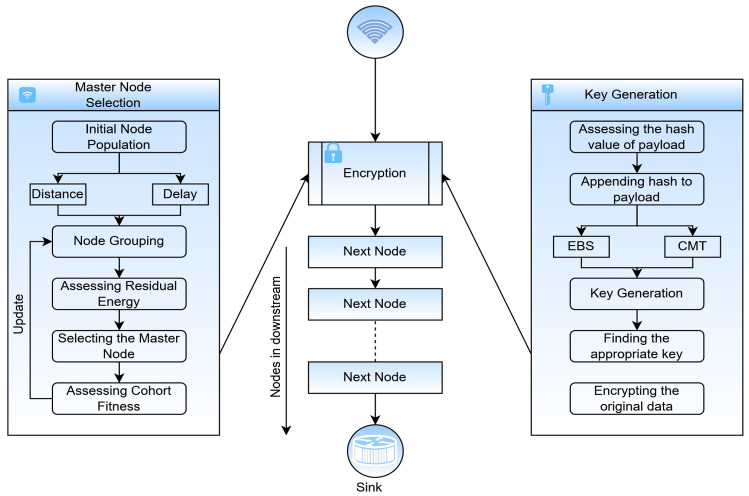
Flow chart of the proposed accumulative security model.

The keys are generated for each round of communication, which would result in computational overhead. However, data security is strongly desired in domains such as healthcare that work with sensitive data. The proposed key generation model is robust to the topological changes, which is a much-desired feature concerning the lifetime of the sensor nodes. The flow chart of the proposed security model is presented in Figure 6, and the corresponding algorithm is presented in Algorithm 1.
**Algorithm 1:** Algorithm of Proposed Security Model
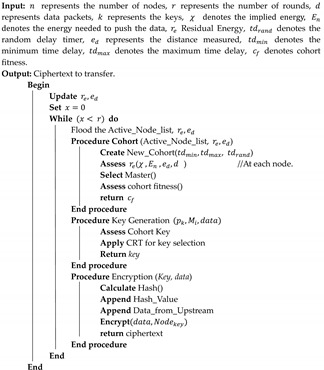


### 4.4. Integration of the Proposed Network Model in AAL

The proposed security model could be deployed in the distributed environment using sensor network technology. The master node is identified based on the available residual energy, and the master node is responsible for encrypting the data and assessing the hash value. The proposed security model is feasible to implement in ambient assisted technology. The sensor devices, controllers, Rf-id readers, and wearable technologies are connected to the trans-receiver, and the data from the transceivers are transferred in an encrypted format. The transceivers with better residual energy in the sensor technology are assumed to be the master, responsible for transferring the data in the secured format. Generally, the transceivers are heterogeneous concerning the applications in which they are deployed. In the current study, for performance analysis, the transceiver nodes are considered homogeneous nodes with divergent residual energy levels. 

## 5. Results and Discussion

**Figure 7:**
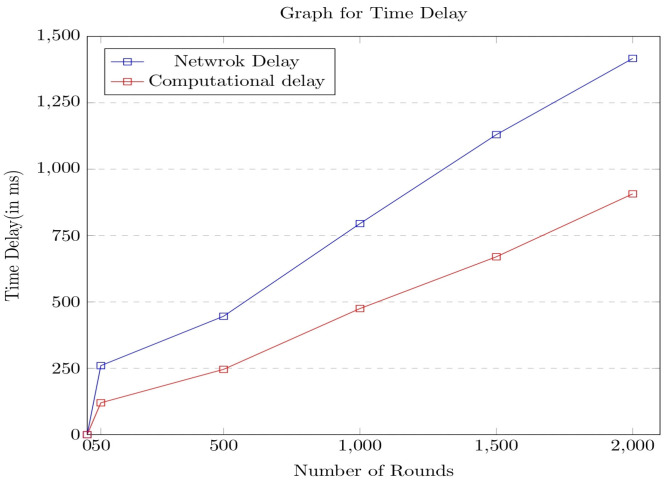
Graph portrays the time delay associated with the proposed security framework.

The efficiency of the proposed encryption model is analyzed concerning the various standard metrics such as network delay, computation delay, and energy consumption. The obtained experimental values concerning the metrics are analyzed across the other contemporary models in the current section of the paper. The experiment is performed for 2000 rounds, and the delay is assessed in milliseconds. Initially, the time delay is measured across the number of rounds of the network life cycle, and the corresponding graph is shown in Figure 7. 

**Figure 8:**
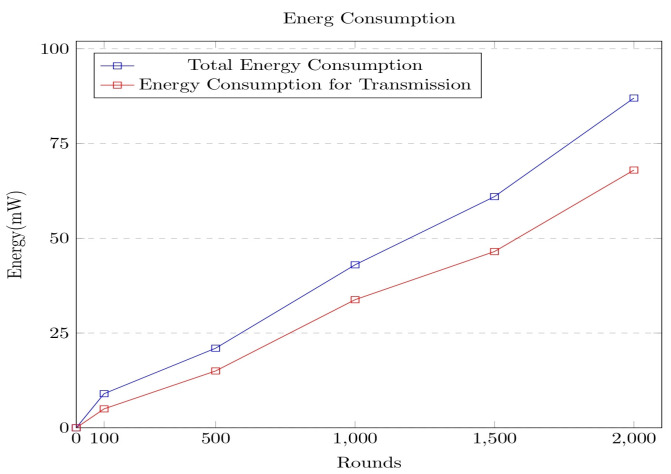
Graph portrays the energy consumption associated with the proposed security framework.

It can be observed from the above graph that the energy consumption has linearly increased over the rounds, as in each round, more data packets arrive from the various sources, resulting in delays at both the network and computational levels. It was clear from the experimental results that the delay incurred by the network is comparatively higher than the computational delay. The model was further evaluated concerning energy consumption over multiple rounds. The corresponding graph is shown in Figure 8. 

**Figure 9:**
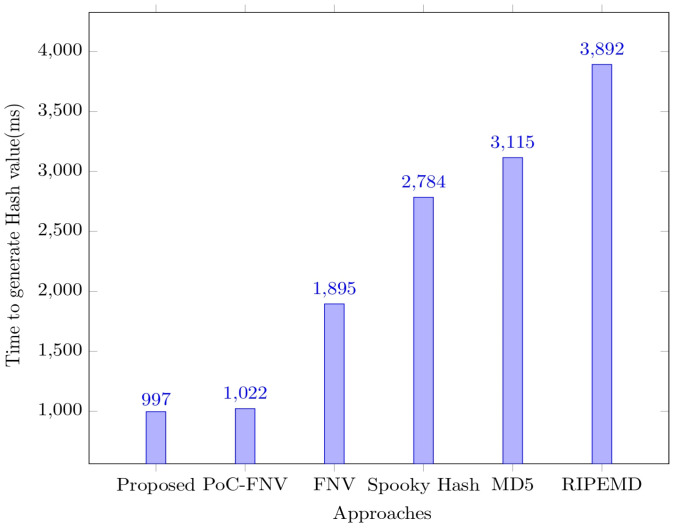
Graph representing the time to generate a hash value.

The energy consumption increases over the rounds, as the number of data packets increases over the rounds, resulting in more energy consumption. The network transmission consumes energy that would linearly increase with the number of rounds. It can be observed from the graph that the energy is not fully utilized after 2000 rounds, which makes it evident about the network’s sustainability. The performance of the secure model framework is further analyzed concerning the time for generation of the hash and the level of security. The robustness of the security mechanism in the proposed model is analyzed with other approaches such as Point-on-Curve-based Fowler–Noll–Vo (PoC-FNV), RACE Integrity Primitives Evaluation Message Digest (RIPEMD), Message Digest Algorithm 5 (MD5), Spooky Hash, and Fowler–Noll–Vo (FNV) [30,31]. The corresponding graph for time consumption to generate the graph is shown in Figure 9.

**Figure 10:**
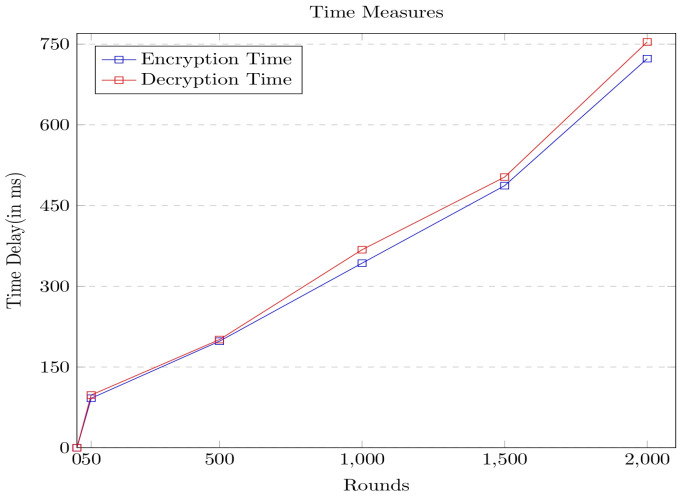
Graph portraying a trade-off between encryption and decryption time.

It can be observed from the above graph that the time taken to generate the hash value used in encrypting the data is comparatively less than the other approaches considered for analysis. The efficiency of the software-driven secure framework is further evaluated concerning the encryption time and the decryption time consumed concerning the number of data packets in the queue. The time measures are presented in Figure 10. 

From the above graph, it can be observed that the decryption time is slightly higher than the encryption time. In the current study, the encryption and decryption times are calculated by considering the network delay at the corresponding round in the network. To better understand the procedure, the notation identifies the total time delay $tt_{d}\;$ for encryption or decryption operation is assessed using the formula shown in Equation (24).

(24) $$tt_{d}=N_{dy}+O_{dy}$$

From the above equation, the notation $N_{dy}$ denotes the network delay and $O_{dy}$ denotes the operation delay, where the operation refers to encryption or decryption. The data size is 6400 bits in the current study, which is constant throughout the evaluation. The data is divided into multiple virtual blocks based on the size of the key for ease of evaluation, as shown in Equation (25). The number of cycles per block is assessed using the formula shown in Equation (26).

(25) $$Number\;of\;Blocks=\frac{Data\;size}{Key\;size}$$

(26) $$Cycles\;per\;Block=\frac{Cycles\;per\;second}{Speed}$$

The cycles per second depend on the clock frequency of the processor. Hence, this largely depends on the machine on which the model is executed. The operation time is assessed using the formula shown in Equation (27).

(27) $$O_{dy}=Number\;of\;Blocks\times Cycles\;per\;Block$$

The performance of the encryption model is further analyzed concerning the entropy value of the encrypted text. The higher the degree of entropy, the greater the system’s complexity. In other words, the information entropy effectively measures the system. The formula for information entropy is measured as shown in Equation (28) [32].

(28) $$e\left(d\right)=\underset{d}{\sum }p\left(d\right)log_{2}\frac{1}{p\left(d\right)}$$

**Figure 11:**
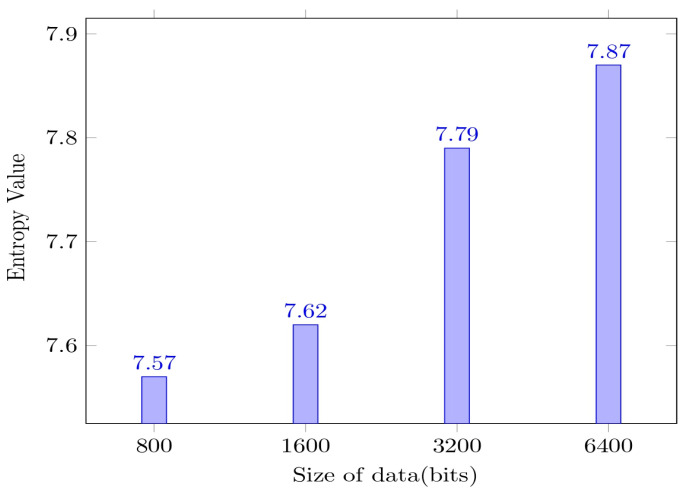
Graph portraying entropy values of the proposed model.

The entropy is measured for data of various sizes. The actual size of data considered in exchanging the data is 6400 bits, entropy value is assessed for variable-size data blocks, and the same is shown in Figure 11.

**Figure 12:**
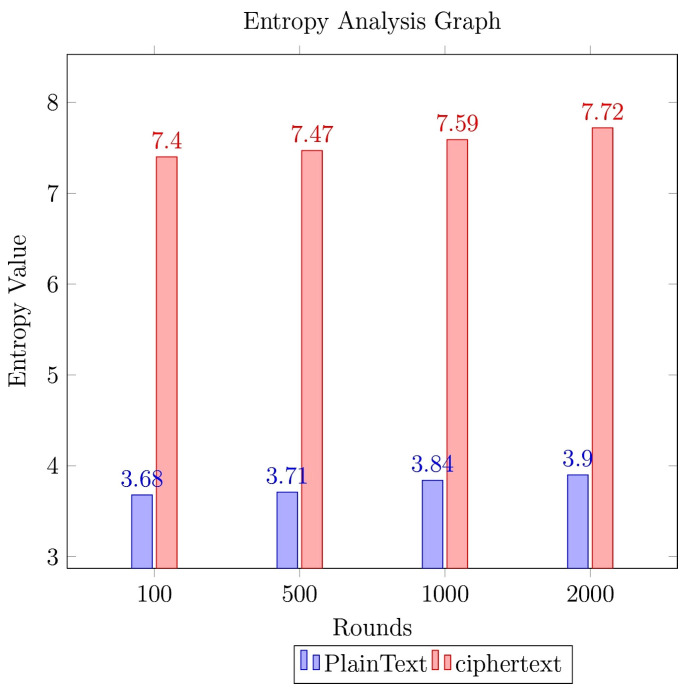
Graph portraying entropy values of plain text and cipher text at divergent rounds.

The figure shows that analyzing the text would be more difficult with more bits in the original data. The higher the entropy value, the more the randomness in the data and the more complex the cipher text. It is desired to have a higher entropy value, to make sure the encryption model is robust. The increase in the data size would significantly impact the entropy value, with a higher number of bits in the payload, which would generate a relatively larger cipher text that would have a better entropy value [33]. The entropy of the plain data and the corresponding cipher text is analyzed at multiple rounds for better comprehension of the study. The corresponding graph for entropy measure is shown in Figure 12.

As can be observed from the graph, the size of the data is retained to be uniform to maintain the consistency of data throughout the analysis; the entropy of the cipher text is experienced to be high at higher rounds, due to the involvement of multiple nodes in communication and the resultant aggregated data. The encryption model would process the data bits received from the upstream, where the data of nodes would be merged to form the block, resulting in better randomness. However, there is a marginal change in the entropy value of the data over the rounds, with a change of 0.32 over 2000 rounds of implementation.

**Figure 13:**
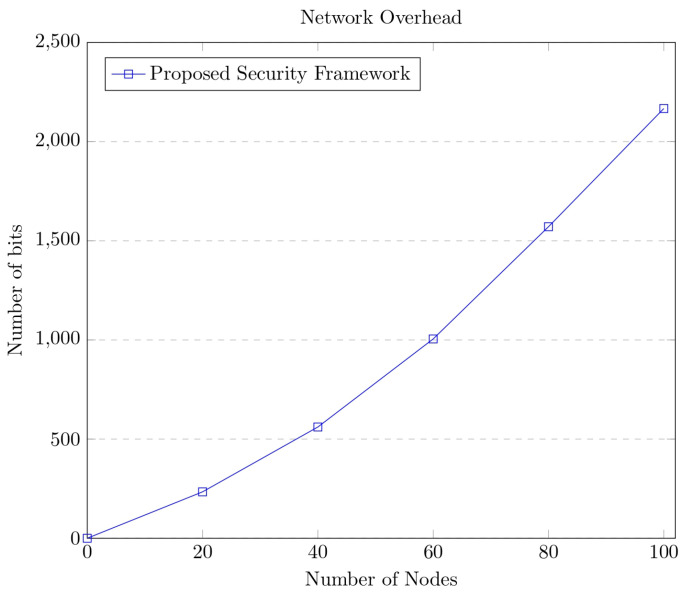
Graph portraying network overhead of the proposed model.

The model may be analyzed over divergent key sizes for better performance analysis. The extra information taken when transferring packets is determined by network overhead. Because increased overhead leverages network performance, overhead substantially influences total network performance. The network overhead concerning the number of bits in connection to the number of devices is shown in Figure 13. 

The experimental findings show that, as the sum of nodes in a network grows, so does the amount of overhead it incurs. Congestion in the network is avoided since the approach does not show an exponential development in network overhead. The performance of the proposed security model for AAT was analyzed over divergent metrics such as energy consumption, network delay, network overhead, and time in generating hash, as well as the tradeoff between encryption and decryption delay. It was observed that the proposed model exhibited a reasonable performance in all the cases considered for evaluating the model’s performance. 

The potential limitation of the current study includes the type of nodes being considered in the evaluation process. Due to the underlying constraints, all of the nodes are considered to be heterogenous, and in real time, the nodes are heterogeneous, so the results and analysis are confined to the limited functionality of the proposed network model. The strength of the security model is proportional to the size of the key used in the encryption process [34]. In the current study, the key size is fixed throughout the analysis, and the model’s performance can be further evaluated against varied key sizes. The crypt analysis concerning any of them is not performed in the current study, which is a potential limitation. 

## 6. Conclusions

The security of data exchanges over the sensor devices in IoT technology is exceptionally important, and the current study focuses on ensuring the security of the more sensitive data in the distributed environment. The data are encrypted at the master nodes to ensure optimal utilization of the energy resources for better network sustainability. The master nodes in each cluster are responsible for data encryption using the data aggregation mechanism and public key cryptosystem. The model’s performance was analyzed concerning the network delay, computational delay, network overhead, energy consumption for transmission, and overall energy consumption. It was observed that the model exhibited a reasonable performance concerning all of the parameters mentioned above. It was observed from the experimental outcome that the model showed a linear growth in energy consumption, network delay, and cryptographic time measured over the rounds with a rise in the overall number of nodes in the network that are involved in communication, which proves there is incremental demand for the resources rather than an exponential growth that would impact the sustainability of the network. The entropy measures prove that the cipher text is complex to perform the crypt analysis by the attackers. The study was limited to the homogeneous sensor nodes responsible for sensing and transmitting the data over the network. Still, in real-time scenarios, the network must work with heterogeneous sensors and network nodes in the environment. Only evaluating the model with homogeneous nodes is a major potential limitation of the current study, as the real-time performance would broadly vary from the simulated environment. 

As the proposed software-defined security model is deployed in the medical setting, it is necessary to evaluate the model with divergent sensor nodes to extensively analyze the efficiency of the model. The security of the model can be further analyzed over the varied key size to analyze the impact of the key size on the confidentiality of the data. Moreover, the aggregation-based data encryption models are suitable for networks with limited nodes. The crypt analysis is one of the pivotal limitations of the current study, which can be addressed in future research on various attacks. Blockchain-based models would be more reliable with networks with more nodes. However, blockchain implementation needs considerable resources, and the wireless sensor network is sensitive to computational resources. The influence of noise over the network delay is not considered in the current study, which is one of the potential limitations of the current study. However, noise has a significant impact on the performance of the model; hence, an in-depth analysis of noise modulation would assist in comprehending the real-time performance of the model. The noise in the channel is the other important aspect, where the noise is maintained to be constant in the current study, but in a real-time scenario, the noise would change across the channel based on divergent factors. The performances at divergent noise levels could be analyzed in future studies. There is great scope for research in designing lightweight security models that could accommodate more nodes and a network with heterogeneous nodes. 

## Figures and Tables

**Table 1:** Specifications of the experimental environment.

Environment Details	Specifications
Simulation area	100 m ×100 m
Location of sink	50 m ×50 m
Total sinks in the network	1
Total nodes in the network	100
Nature of nodes	Stationary
The energy at node (round 0)	100 mW
Message length (slave to master)	2800-bits
Message length (master to sink)	6400-bits
Number of rounds of simulation	2000
Limit of transmission distance	30 m
Intra-cluster routing	Single Hop
Radio range	100 m
Traffic category	Multicast constant bit ratio
Protocol	IEEE 802.11

**Table 2:** List of notations used in the current section.

Notation	Description
$A_{data}$	Actual Data
$HV_{data}$	The hash value of the data
$DT\left(f_{dA}\right)$	Data transfer from the node A
$DT\left(f_{dB}\right)$	Data transfer from the node B
$DT\left(f_{dC}\right)$	Data transfer from the node C
$DT\left(f_{dD}\right)$	Data transfer from the node D
$Acc\left(A\right)$	Accumulated data at node A
$Acc\left(B\right)$	Accumulated data at node B
$Acc\left(C\right)$	Accumulated data at node C
$E\_Acc_{data}$	Encrypted Accumulated data
$k_{A,B}$	Key to transfer the data from A to B
$k_{A,C}$	Key to transfer the data from A to C
$k_{A,D}$	Key to transfer the data from A to D
$k_{B,C}$	Key to transfer the data from B to C
$k_{B,D}$	Key to transfer the data from B to D
$k_{C,D}$	Key to transfer the data from C to D
$\;C_{key}$	Cohort’s key
$\;I_{m}$	Index of Master nodes
$p_{key}$	Private key
$c_{key}$	Cohort’s key
$m_{key}$	Master node’s key

## Data Availability

Not applicable to the current study.
